# Report of Some Cases from the Eye and Ear and Throat Clinic

**Published:** 1879-04-20

**Authors:** A. W. Calhoun

**Affiliations:** Georgia


### Southern Medical Record.

EDITORS:

T. S. POWELL, M.D. W. T. GOLDSMITH, M.D. R. C. WORD, M.D

*R. C. WORD, M.D., Managing Editor.*

☞All Communications and Letters on Business connected with the RECORD must be addressed to the Managing Editor.

VOL. IX. ATLANTA, GA., APRIL 20, 1879. NO. 4.

### ORIGINAL AND SELECTED ARTICLES.

### *REPORT OF SOME CASES FROM THE EYE AND EAR AND THROAT CLINIC.*

BY PROF. A. W. CALHOUN, OF GEORGIA.

CASE I.—*Blepharitis.*

G. H., white girl, aged six years, of Atlanta, has good general health, but the edges of the lids of both eyes are thickened and covered with a scaly formation resembling dandruff of the scalp. The conjunctiva is also slightly but constantly irritated. This condition of things is of one year's duration and is greatly increased by every little cold or exposure to wind, smoke, etc. The following was prescribed :

℞ Hyd. ox flav.............................. gr ij

Unguent emollient......................... ℥ j

M. et faciat unguent.

S. Rub a small piece into edges of lids every night after scratching off the scales.

℞ Zinci sulph................................ gr j
Aluminis.................................. gr. ij
Morphiæ sulph............................. gr. j
Aqua distil.,.............................. ℥ j

S. Drop few drops into eye three times daily.

*Note.*—The above treatment is effecting a rapid cure of the disease.

CASE 2.—*Chronic suppurative Inflammation* (*Otorrhœa*) *of the left Ear.*

Mr. W. R. G. (white), of Stone Mountain, Ga., aged 19 years, had scarlet fever when 4 or 5 years old, as a result of which he has had a constant and profuse offensive discharge from the left ear, diminishing the hearing to a very decided degree. Examination of the ear discovers the fact that there is a large perforation of the drum membrane. The treatment was as follows :

℞ Tinct. iodini........................................... ʒ j

Iod. potassii........................................... ʒ jss

Glycerini.................................................. ℥ ij

S. Pour, lukewarm, three times daily into ear, after syringing it. Also :

℞ Aluminis pulv.................................. ʒ ij

Licopodii........................................... gr. xx

Rub into a fine powder.

S. Pack the ear full every second night, after syringing and drying it, and leave in for 12 to 24 hours.

With this treatment alone the patient was in four weeks' time cured of a loathsome disease that had existed nearly fifteen years.

CASE 3.—*Purulent Inflammation and closure of the tear passage* (*dacrocystitis.*)

Mr. F. M., (white), of Georgia, aged 54, has had granular lids for a great number of years, as a result of which there appeared inflammation of the lachrymal sac and the nasal duct of the left eye, filling the first with matter, and causing stricture of the latter. By pressing upon the skin over the sac, a quantity of pus would flow out upon the conjunctiva of the lower lid. As can be imagined, this state of things was producing a number of obvious evils. The lower canaliculus was dilated by means of small wire-probes, until the blunt-pointed knife (used for this purpose) could be introduced. The canaliculus was then slit at into the lachrymal sac, and the knife carried on down to the very bottom of the nasal duct. A Bowman's silver-probe No. 8 was daily introduced for five or six weeks and allowed to remain ten minutes at a time. At the end of this time, the patient had himself learned to introduce the probe, and was dismissed, cured, with directions to continue the probing at intervals of a week to ten days, for some time to come.

In this case, there was an inturning of the lashes of the upper lids against the balls (entropion) which was rapidly cured by cutting out an elliptical piece of skin from each upper lid, and bringing the edges of the wound together by silk sutures. This caused the edges of the lids and the lashes to permanently turn outwards in their natural position.

CASE 4.—*Gonorrhœal Ophthalmia, resulting in Corneal Staphyloma of one Eye and Central Corneal Opacity in the other, with blindness in both—Operations and Restoration to Sight.*

J. T., (colored), of Crawfordville, Ga., aged 23, had gonorrhœal ophthalmia in both eyes, the result of the direct transmission of the virus from the penis to the conjunctiva. Most violent inflammation ensued, resulting in complete destruction of the left cornea and the central portion of the sight. During the healing process, the front part of the left ball began to protrude forward and continued to grow till it extended out between the lids to such a degree as to prevent their closure, constituting complete staphyloma. The right cornea was ulcerated in front of the pupil, so that when it healed, a large white cicatrix completely cut off the entrance of light into the interior of the ball. He was thus totally blind in both eyes.

The protruding portion (staphyloma) of the left ball was removed by making Critchett's operation, which healed nicely and left a well-formed stump, upon which an artificial eye can be worn at any time.

The lower portion of the right cornea being clear, a large iridectomy (artificial pupil) was made, thus affording a window through which the light could enter into the eye. This was eminently successful, giving him enough light to enable him to return home and continue his avocation (farming) with tolerable ease.

More or less thorough destruction of the cornea always takes place in gonorrhœal ophthalmia, unless the disease is vigorously treated from the very beginning.

